# Generalized anaphylactic reaction after ingestion of shrimp in patient with bronchial asthma

**DOI:** 10.1186/2045-7022-3-S3-P160

**Published:** 2013-07-25

**Authors:** N Ukleja-Sokolowska, E Gawronska-Ukleja, M Zbikowska-Gotz, Z Bartuzi

**Affiliations:** 1 Department and Clinic of Allergy, Clinical Immunology and Internal Diseases, Collegium Medicum in Bydgoszcz, Nicolaus Copernicus University, Torun, Bydgoszcz, Poland

## Background

Nowadays in countries all over Europe more and more people consume shrimps and other types of sea food. Consumption of even one or two shrimps can induce anaphylactic reaction in patient with allergy.

In this work our aim is to present a case of patient admitted to the Department and Clinic of Allergy, Clinical Immunology and Internal Diseases to diagnose and confirm allergy to shrimp.

## Methods

Patient, male, 36 years old, after ingestion of shrimp experienced swallowing of eyes, generalized urticaria and blisters on the whole body and severe dyspnea. Patient was treated because of allergy to house dust mite, with nasal blockade as the main symptom. In his family history there were no allergic diseases.

Patient had wide range of diagnostics performed: skin prick test (SPT) with shrimp allergen extract (Stallergen), skin prick test with basic panel of food and inhaled allergens using standardized extract (Allergopharma). Immunological diagnostic was perfomed using EUROLineScan automatic method. To objectively confirm allergy to shrimp patient had prick by prick test with Black Tiger shrimp (*Penaeus monodon*) and with shrimp prepared in liquid nitrogen in our laboratory.

Also we performed spirometry with histamine provocation.

Next we determined the level of specyfic IgE against cod and Dermatophagoides pteronyssinus and Dermatophagoides farinae.

## Results

Based on the result of spirometry we diagnosed chronic mild bronchial asthma.

SPT results: positive test with Dermatophagoides pteronyssinus, Dermatophagoides farinae and Artemisia vulgaris. The tests were negative with all food allergen extracts including cod and shrimp.

Prick by prick test with native shrimp and cod was strongly positive (6mm blister, positive control 4mm, negative control - 0), prick by prick test with shrimp in liquid nitrogen was positive (shrimp 5/9 mm, cod 5/6 mm).

The level of specific IgE against cod was below 0,35 IU/ml (negative), Dermatophagoides pteronyssinus 19,83 (IV class) Dermatophagoides farinae 2,5 (II class).

## Conclusion

Native prick by prick test enabled to objectively diagnose of allergy to shrimp in described patient, whereas standardized allergen extract did not allow to set the diagnosis.

We can suspect that in this case allergy to shrimp was gathered with cross reactivity with house dust mite tropomyosin.

To confirm cross reactivity reaction it is necessary to carry out a research with recombinant antigen using techniques such as RAST test or Immunocap isac.

## Disclosure of interest

None declared.

